# Durvalumab with or without tremelimumab for patients with recurrent or metastatic squamous cell carcinoma of the head and neck: a systematic review and meta-analysis

**DOI:** 10.3389/fimmu.2023.1302840

**Published:** 2024-01-17

**Authors:** Xiao Han, Haidong Zhang, Kai Sun, Jing Li, Wanjuan Wu, Kai Liu, Zhenkun Yu

**Affiliations:** ^1^ Department of Otolaryngology-Head and Neck Surgery, BenQ Medical Center, The Affiliated BenQ Hospital of Nanjing Medical University, Nanjing, Jiangsu, China; ^2^ The Nanjing Medical Key Laboratory of Laryngopharynx and Head and Neck Neoplasm, BenQ Medical Center, The Affiliated BenQ Hospital of Nanjing Medical University, Nanjing, Jiangsu, China

**Keywords:** durvalumab, immunotherapy, meta-analysis, recurrent or metastatic HNSCC, tremelimumab

## Abstract

**Objective:**

Head and neck squamous cell carcinoma (HNSCC) ranks as the sixth most prevalent cancer worldwide, significantly impacting patients’ quality of life. Immune checkpoint inhibitors (ICI) have been employed in the treatment of recurrent/metastatic (R/M)-HNSCC patients. This meta-analysis aims to assess the efficacy and safety of durvalumab monotherapy compared to the combination of durvalumab and tremelimumab in R/M-HNSCC patients.

**Methods:**

Relevant studies were systematically searched in PubMed, Embase, and Cochrane Library databases. All articles comparing durvalumab monotherapy with the combination with durvalumab and tremelimumab in R/M-HNSCC treatment were included. Additionally, the references of identified studies were screened if necessary.

**Result:**

A total of 1298 patients from three studies comparing durvalumab with durvalumab and tremelimumab in treating R/M-HNSCC were include in this meta-analysis. Our findings revealed no significant difference in objective response rate (ORR) [odds ratio (OR): 1.15, 95% confidence interval (CI): 0.85 to 1.56, P = 0.36] and disease control rate (DCR) (OR=1.08, 95%CI: 0.86 to 1.37, P = 0.51). Similar outcomes were observed in overall survival (OS), progression-free survival (PFS), and duration of response (DoR). Regarding safety, there was no significant difference in the incidence of treatment-related adverse events (trAEs) between the two groups (OR=1.26, 95%CI: 0.81 to 1.94, P = 0.30). However, patients treated with the combination therapy exhibited a higher incidence of grade 3-4 trAEs (OR=1.93, 95%CI: 1.36 to 2.73, P = 0.0002) and a greater likelihood of discontinuing treatment due to trAEs (OR=2.07, 95%CI: 1.12 to 3.85, P = 0.02). There was no significant difference in the occurrence of severe trAEs leading to death (OR=1.36, 95%CI: 0.47 to 3.96, P = 0.57).

**Conclusion:**

This meta-analysis suggests that R/M-HNSCC patients receiving the combination of durvalumab and tremelimumab may achieve comparable outcomes in terms of ORR, DCR, OS, PFS, and DoR, without significant differences. However, the combination therapy is associated with a higher incidence of grade 3-4 trAEs and an increased likelihood of treatment discontinuation due to trAEs. These findings highlight the need for cautious consideration of the combination of durvalumab and tremelimumab in R/M-HNSCC patients, which should be further evaluated in high-quality studies.

## 1 Introduction

Head and neck squamous cell carcinoma (HNSCC) is the sixth most prevalent cancer worldwide, with its incidence steadily increasing (1, 2). Despite advancements in HNSCC diagnosis and treatment over the past three decades, a significant proportion of patients experience local recurrence and/or distant metastasis with poorer prognosis, and receive palliative and systemic care (3). Currently, novel immunotherapies, such as immune checkpoint inhibitors (ICI) including programmed cell death protein (PD-1) and anti-programmed cell death 1 ligand 1 (PD-L1), are being used for the treatment of patients with recurrent/metastatic (R/M)-HNSCC (4–6). Numerous clinical trials have demonstrated the favorable anti-tumor effects of ICI therapy in patients with R/M-HNSCC (7–10).

Durvalumab, a fully human immunoglobulin G1k monoclonal antibody, is a potent antagonist of PD-L1 function, blocking its interaction with PD-1 and CD80, thereby overcoming inhibition of T cell activation (11, 12). Durvalumab received accelerated approval in May 2017 for the treatment of patients with locally advanced or metastatic uroepithelial cancer (13). Subsequently, it has also shown clinical activity in other solid tumors, such as non-small cell lung cancer (NSCLC) (14), esophageal squamous cell carcinoma (15), and HNSCC (16). In a Phase I/II cohort study in patients with R/M-HNSCC, durvalumab demonstrated a manageable safety profile and durable anti-tumor activity (16). Tremelimumab, a fully human IgG2 monoclonal antibody with high specificity for cytotoxic T-lymphocyte-associated protein 4 (CTLA4), antagonizes the binding of CTLA4 to B7 ligand, thereby enhancing T cell activation (17). Combining dual-targeted immunotherapy with a PD-1/PD-L pathway blocker and a CTLA-4 blocker provides more potent antitumor activity than either drug alone, resulting in improved efficacy (18, 19). In the treatment of patients with R/M-HNSCC, the combination of durvalumab and temlimumab produced clinical benefit, but it remains uncertain whether combination therapy is superior to durvalumab monotherapy (7, 20).

Although ICIs have been used as immunotherapies for the treatment of many cancers, they can lead to a range of long-term side effects (21). Specific side effects exhibited by patients receiving immunotherapy are known as immune-related adverse events (irAEs) (22). IrAEs can affect various organ system, including the skin, gastrointestinal tract, respiratory system, endocrine system, and liver (23). Furthermore, without timely intervention, irAEs can lead to termination and failure of the patient’s treatment, or even death (24). Studies have yielded different conclusions regarding whether side effects are higher with durvalumab monotherapy or durvalumab in combination with tremelimumab (7, 25–29).

Therefore, we conducted this systematic review and meta-analysis to evaluate the efficacy and safety of durvalumab monotherapy as well as durvalumab in combination with tremelimumab in patients with R/M-HNSCC.

## 2 Method

### 2.1 Search strategy

The articles were searched in PubMed (until August 2023), Embase (until August 2023), and Cochrane Library databases (until August 2023) following Preferred Reporting Items for Systematic Reviews and Meta-analyses (PRISMA) criteria (30). The search keywords were applied as follows: durvalumab, tremelimumab, HNSCC, CTLA-4, PD-L1, and immunotherapy. The exclusion criteria were listed: letters, comments, review articles, qualitative studies, and non-controlled articles. All the searched articles were selected through reading titles, abstracts, and full text by three authors. These studies were also reviewed by authors to confirm the availability and identify additional relevant articles.

### 2.2 Quality assessment

All included studies were RCTs, which were evaluated by the Cochrane Handbook (31). The bias of studies was presented as following the quality assessment criteria: (+) low risk of bias; (?) moderate risk of bias; (-) high risk of bias including selection bias, performance bias, detection bias, attrition bias, and reporting bias. All authors resolved their differences of opinion on the classification through discussion.

### 2.3 Data extraction

The data extraction was also finished by two different authors, and checked by a third one. A series of valuable information was collected from all included studies as follows: main authors, the publication year of study, NCT ID of trial, treatment method, design of the trial, the time frame of trial, patients age, patient number in each cohort, the dosage of each treatment and objective response rate (ORR), disease control rate (DCR), progression-free survival (PFS), overall survival (OS), duration of response (DoR), treatment related adverse events (trAEs).

### 2.4 Statistical and meta-analysis

Review Manager software (RevMan, version 5.3.0, Cochrane Collaboration) was used to statistically analyze all collected data and draw the creation of forest plots for this meta-analysis (32). The dichotomous data was evaluated by odds ratios (ORs) with 95% confidence intervals (95% CIs) (33). If the I^2^ statistic was less than 50%, the fixed-effects model was applied as the study was deemed to be homogeneous; on contraries, the randomized-effects model was used. If the P value was less than 0.05, the results were considered to indicate statistical significance.

## 3 Result

### 3.1 Characteristics of included studies

**Figure 1:**
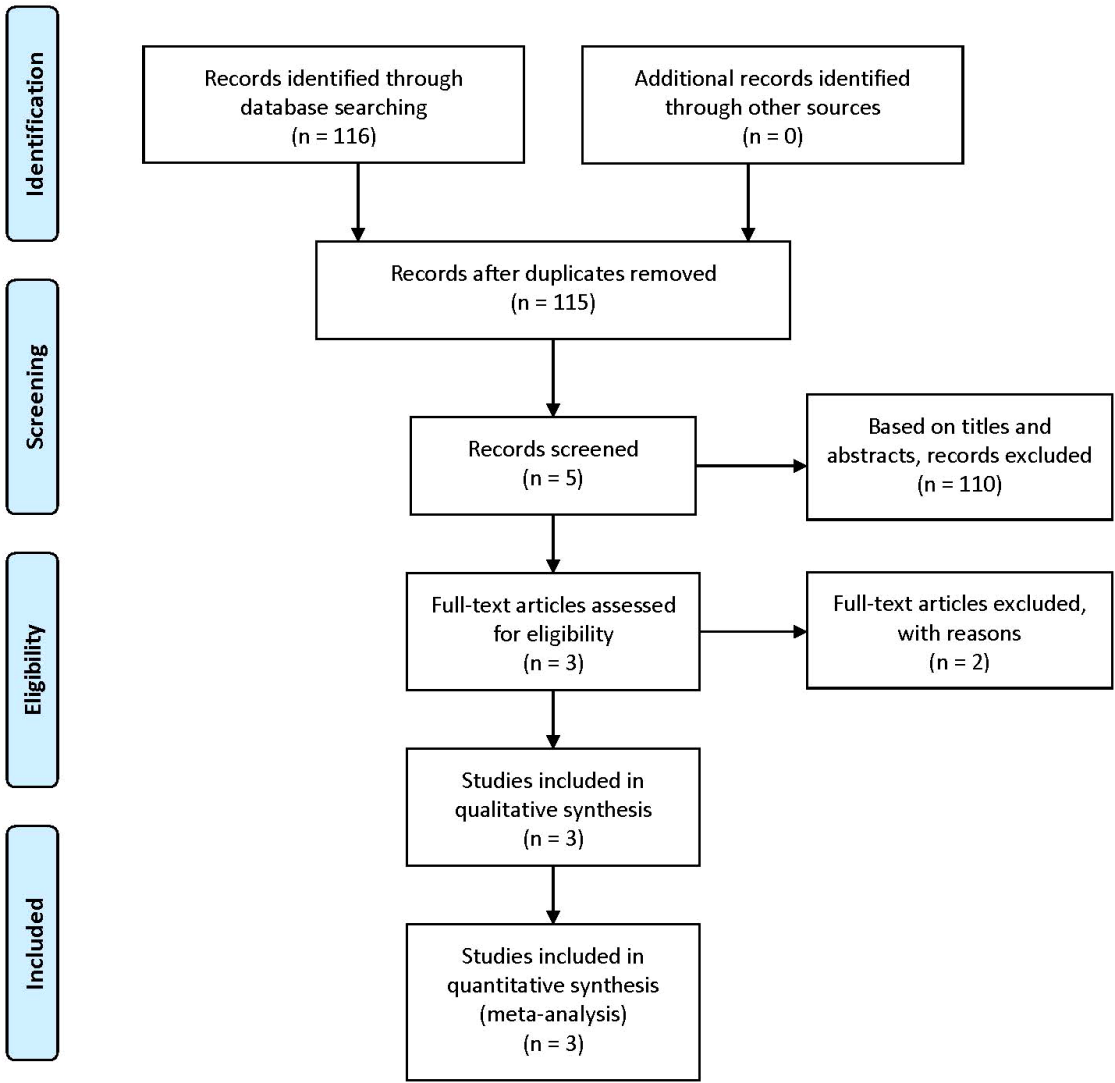
Flow chart of the selection process.

According to our inclusion keywords, we finally found 116 articles in PubMed, Embase, and Cochrane databases. After removing the same articles, 115 articles were read by three different authors. We only retained 5 articles based on titles and abstracts. Then we read full-text articles, 2 articles were excluded because of lack of useful information. Finally, only 3 articles including 1298 patients were included in our meta-analysis (7, 20, 34). The details of the selection procedure are presented in **Figure 1**, and the characteristics and baseline of included studies are shown in **Table 1**.

**Table 1:** Fundamental characteristics of the chosen clinical trials.

Study (years)	NCT ID	Treatment	Design	Follow-up	Age (year)	Patients	Dosage
Ferris et al. (2020)	NCT02369874	Durvalumab+tremelimumab Durvalumab	A randomized, open-label phase III study	5 November 2015 to 21 July 2017	61 59	247 240	Durvalumab+tremelimumab (20 mg/kg durvalumab every 4 weeks plus 1mg/kg tremelimumab every 4 weeks) for 4 cycles followed by durvalumab (10 mg/kg every 2weeks) Durvalumab monotherapy (10 mg/kg every 2 weeks)
Psyrri et al. (2023)	NCT02551159	Durvalumab+tremelimumab Durvalumab	A randomized, open-label phase III study	Q4 of 2015 To Q2 of 2017	61 62	413 204	Durvalumab+tremelimumab (1500mg durvalumab every 4 weeks plus 75mg tremelimumab every 4 weeks) for 4 cycles followed by durvalumab (1500mg every 4 weeks) Durvalumab monotherapy (1500mg every 4 weeks)
Siu et al. (2019)	NCT02319044	Durvalumab+tremelimumab Durvalumab	A randomized, open-label, multicenter, global phase 2 study	15 April, 2015 to 16 March 2016	62 62	133 67	Durvalumab+tremelimumab (20 mg/kg durvalumab every 4 weeks plus 1mg/kg tremelimumab every 4 weeks) for 4 cycles followed by durvalumab (10 mg/kg every 2weeks) Durvalumab monotherapy (10 mg/kg every 2 weeks)

### 3.2 The quality of eligible studies

**Figure 2:**
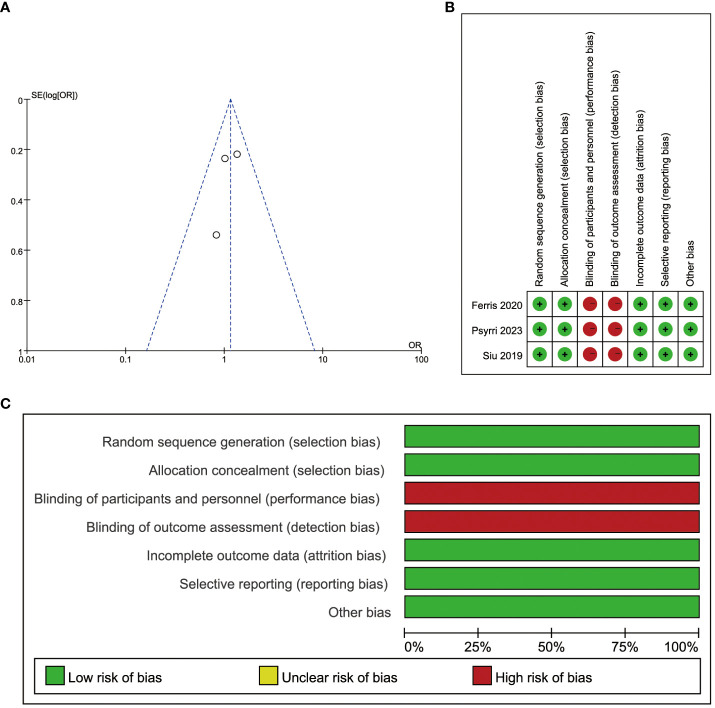
Risk of bias. **(A)** Publication bias assessment for each clinical study included. Dots: included studies; X-axis: odds ratios (ORs); Y-axis: the standard error of the logarithm of the effect size; **(B)** Evaluation of each risk of bias item for each randomized clinical study included. ‘Green + ‘: low risk, ‘red -’: high risk; **(C)** Presentation of each risk of bias item as percentages across all randomized clinical studies included.

All included articles were RCTs comparing durvalumab with or without tremelimumab in patients with recurrent or metastatic head and neck squamous cell carcinoma. Two of the three articles were open-label phase III study, and one was a phase II randomized clinical trial. The summary of the risk of bias is presented in **Figure 2**.

### 3.3 Efficiency

#### 3.3.1 ORR

**Figure 3:**
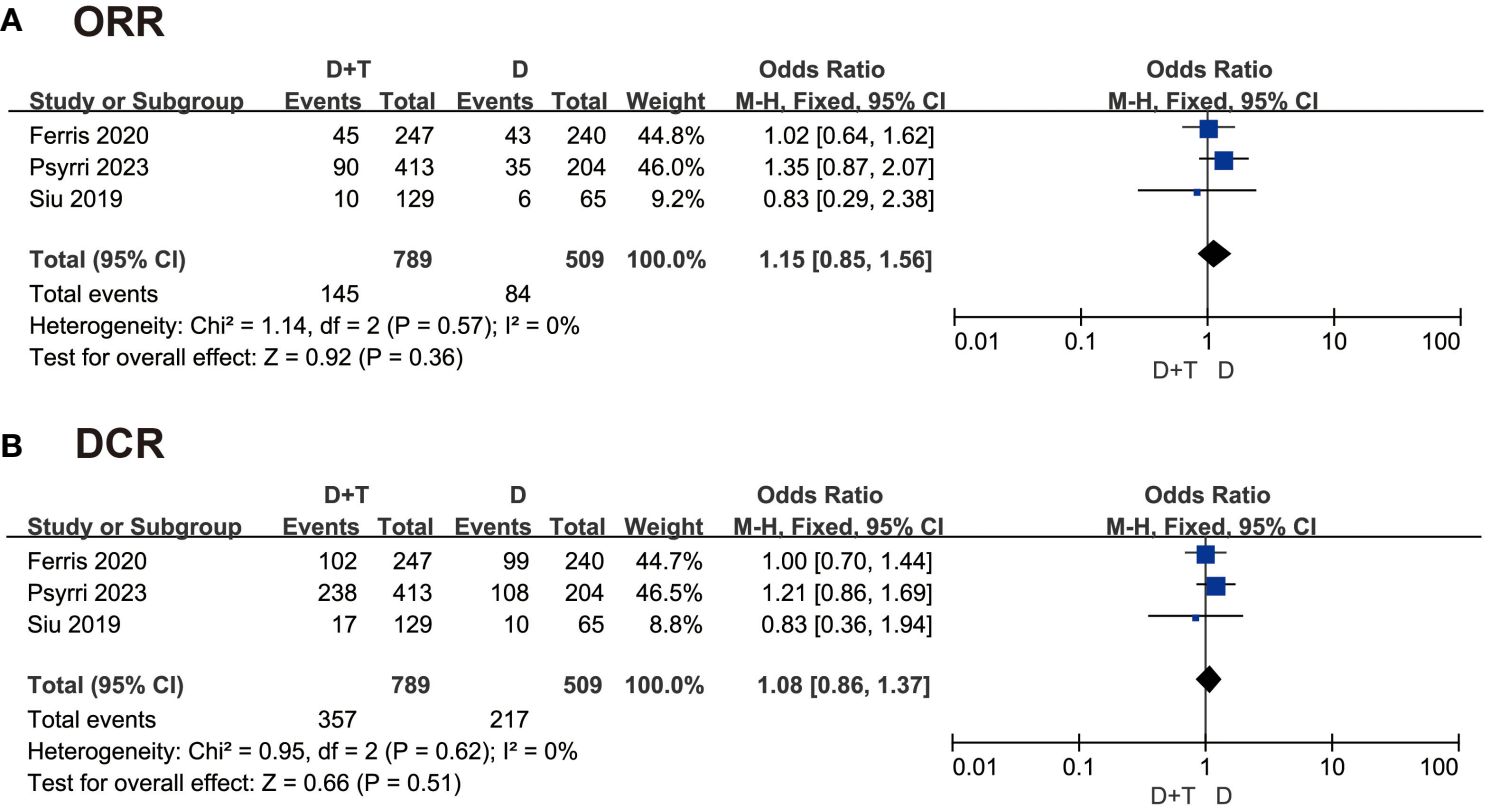
Forest plot comparing **(A)** objective response rate (ORR) and superiority of **(B)** disease control rate (DCR) in patients with recurrent/metastatic head and neck squamous cell carcinoma (R/M-HNSCC).

The included studies were all gettable in objective response rate (ORR). The ORR was patients becoming complete response and partial response in the same group. About 145 patients became complete response and partial response in 789 patients treated with durvalumab plus tremelimumab, and 84 patients became objective response in 509 patients treated with durvalumab. The fixed-effects model was selected as the P value was 0.57 in heterogeneity analysis. The odds ratio (OR) was 1.15 in this group, and the 95% confidence interval (CI) was 0.85 to 1.56 (P = 0.36) (**Figure 3A**). The result indicated that the durvalumab plus tremelimumab could get better ORR, where there was no significant difference.

#### 3.3.2 DCR

The disease control rate (DCR) was the rate of patients becoming complete response, partial response, and stable response in the same group. A total of 1289 patients were included in this analysis. The fixed-effects model was chosen to analyze these data because of P > 0.05. The OR was 1.08 in this group, and the 95% CI was 0.86 to 1.37 (P = 0.51) (**Figure 3B**). This result also suggested that there was no significant difference between the two groups in DCR.

#### 3.3.3 OS, PFS, and DoR

The three included articles described the OS, PFS, and DoR between two groups, which was detailed in **Table 2**. In PFS, Psyrri et al. reported that the median PFS was 2.8 months in durvalumab plus tremelimumab and durvalumab. Siu et al. reported median PFS in durvalumab plus tremelimumab was 2.0 months, and 1.9 months was in durvalumab. The durvalumab plus tremelimumab could not promote PFS significantly. In OS, two articles reported that durvalumab monotherapy was better than durvalumab plus tremelimumab in patients regardless of PD-L1 expression. But two studies described that OS was better in durvalumab plus tremelimumab than durvalumab monotherapy in patients who PD-L1 statue was low/negative. In the survival at 12 months, Psyrri et al. and Siu et al. showed better efficiency in durvalumab plus tremelimumab, whereas Ferris et al. showed the opposite result. In survival at 18 and 24 months, the results of two articles showed that durvalumab monotherapy was better than durvalumab plus tremelimumab. In the duration of response (DoR), all three studies showed that durvalumab monotherapy had better duration than durvalumab plus tremelimumab.

**Table 2:** Overall survival (OS), progression-free survival (PFS), and duration of response (DoR) comparison between the two groups in the three included studies.

Study	Groups	PFS Median months, (95% CI)	OS				DoR Median months, (95% CI)
			Median months, (95% CI)	Survival at 12 months, % (95% Cl)	Survival at 18 months, % (95% Cl)	Survival at 24 months, % (95% Cl)	
Ferris et al. (2020)	Durvalumab+tremelimumab	NA	6.5 (5.5–8.2)	30.4 (24.7–36.3)	21.0 (15.9–26.5)	13.3 (8.9–18.6)	7.4 (3.6–14.8)
	Durvalumab		7.6 (6.1–9.8)	37.0 (30.9–43.1)	25.4 (19.9–31.3)	18.4 (13.3–24.1)	12.9 (6.9–21.0)
Psyrri et al. (2023)	Durvalumab+tremelimumab	2.8 (2.0-2.8)	9.9 (8.9-11.9)	46.5 (41.6-51.2)	30.7 (26.3-35.2)	22.9 (18.9-27.0)	9.2 (6.0-19.6)
	Durvalumab	2.8 (2.0-2.8)	10.7 (9.6-12.2)	42.8 (35.9-49.5)	31.2 (24.9-37.7)	24.7 (18.9-30.8)	11.9 (4.6-17.8)
Siu et al. (2019)	Durvalumab+tremelimumab	2.0 (1.9-2.1)	7.6 (4.9-10.6)	37	NA	NA	9.4 (4.9-NA)
	Durvalumab	1.9 (1.8-2.8)	6.0 (4.0-11.3)	36			NA

NA, not available.

These results could imply that the combination therapy did not show better efficiency than durvalumab monotherapy in recurrent or metastatic HNSCC.

### 3.4 Safety

#### 3.4.1 TrAE

**Figure 4:**
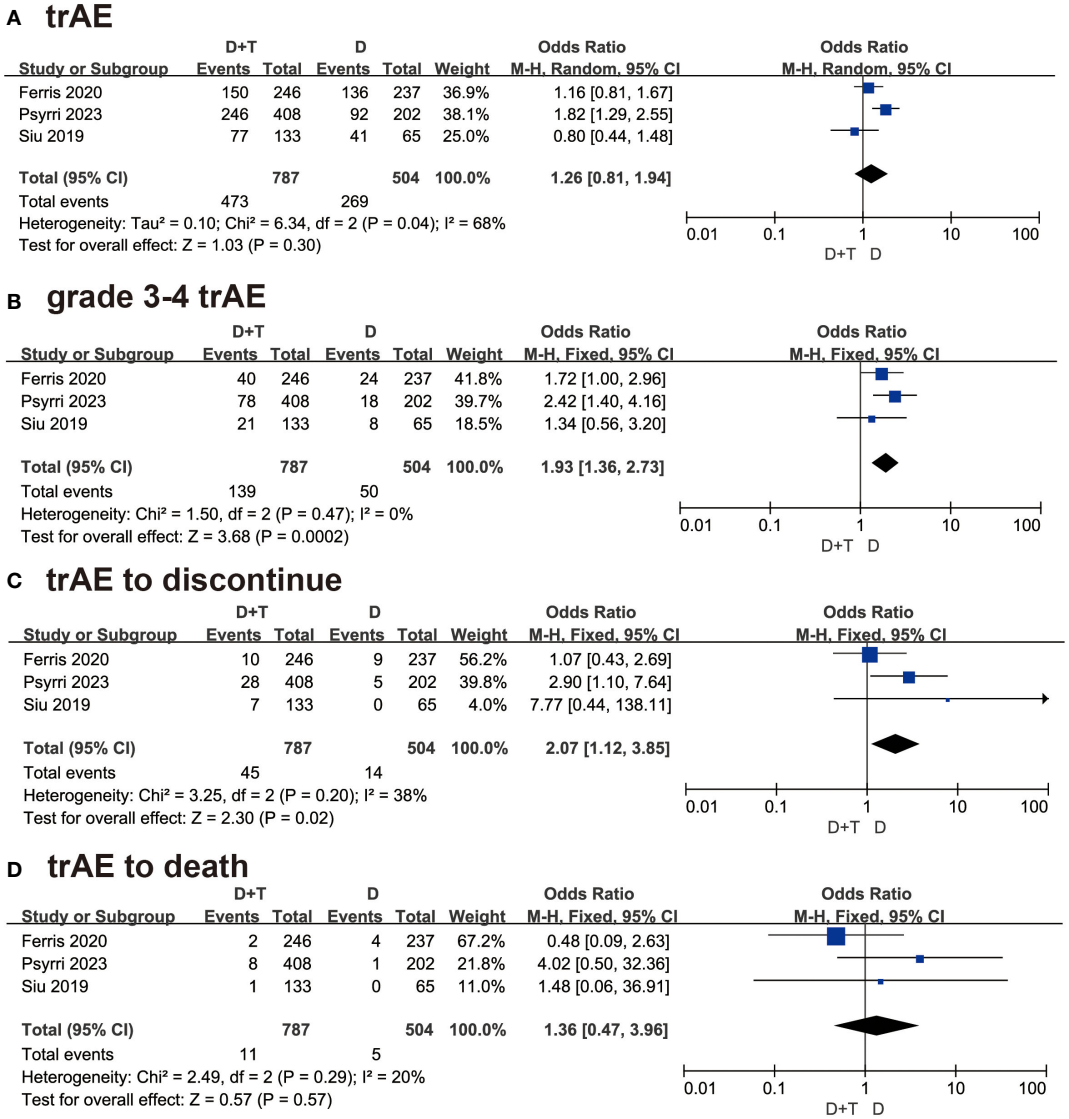
Forest plots illustrating the result of **(A)** treatment-related adverse events (trAEs), **(B)** grade 3-4 trAEs, **(C)** trAE leading to treatment discontinuation, and **(D)** trAEs resulting in death.

A total of 1291 patients in these three studies reported treatment-related adverse events (trAE). As P < 0.05, the random-model effect was selected. The OR was 1.26 in this group, and the 95% CI was 081 to 1.94 (P = 0.30) (**Figure 4A**). The result suggested that the combination of durvalumab plus tremelimumab was similar safety to durvalumab monotherapy.

#### 3.4.2 Grade 3-4 trAE

All three studies detailed the data of grade 3-4 trAEs. About 1291 patients which 787 patients were in combination therapy and 504 patients were in the durvalumab group, were included in this analysis. As P > 0.05, the fixed-effect model was selected. The OR was 1.93 with 95% CI (1.36 to 2.73 (P=0.0002) (**Figure 4B**). The result suggested that patients treated with durvalumab plus tremelimumab had more grade 3-4 adverse events than durvalumab monotherapy.

#### 3.4.3 TrAE to discontinue

Three studies involving 1291 patients reported trAE to discontinue. As P > 0.05, the fixed-effect model was chosen. The OR was 2.07 with 95% CI (1.12 to 3.85) (P=0.02) (**Figure 4C**). This result meant that patients treated with durvalumab plus tremelimumab refused continuous treatment due to trAE more frequently.

#### 3.4.4 TrAE to death

In the trAE to death analysis, the result concluded that patients treated with durvalumab plus tremelimumab died due to trAE were similar to durvalumab monotherapy [OR = 1.36, 95% CI (0.47, 3.96), P = 0.57] (**Figure 4D**).

## 4 Discussions

In the tumor microenvironment (TME), the overexpression of specific inhibitory immune checkpoints, in conjunction with immune checkpoint ligands, can induce T-cell dysfunction or apoptosis and promote tumor immunosuppression (4, 35). ICIs block inhibitory immune checkpoint pathways to reactivate the immune response against cancer and enhance immune system activity (22). Although ICIs have shown promise for durable long-term survival in patients with R/M-HNSCC in recent years (36–38), the optimal treatment approach for R/M-HNSCC is still under investigation.

CTLA4 expression is rapidly upregulated upon T cell activation and binds to B7 molecules with higher affinity than CD28 (39, 40), inhibiting T cell activation and actively transmitting inhibitory signals to T cells (41). PD-1 is also expressed on activated T cells and, when bound to its ligands PD-L1 and PD-L2, promotes T cell dysfunction, apoptosis, and exhaustion (42). Although the CTLA-4 and PD-1 pathways have distinct mechanisms, the combined blockade of both pathways reduces immunosuppression and promotes inflammation in the tumor microenvironment (18). In a phase I/II study, the combination of durvalumab and tremelimumab demonstrated better efficacy than the durvalumab monotherapy regimen in patients with unresectable hepatocellular carcinoma, showing a more encouraging benefit-risk profile (43).

Based on our analysis of existing studies, the results suggest that combination therapy did not exhibit superior efficacy compared to durvalumab monotherapy in R/M-HNSCC, which differs from another previous study (44). However, overall, patients treated with combination of durvalumab and tremelimumab achieved a higher ORR than those treated with durvalumab monotherapy alone, although the difference was not statistically significant. The DoR was also longer with combination therapy than with monotherapy. Additionally, the response to ICI therapy is associated with tumor-infiltrating lymphocytes (45–47). Some of the patients included in this study may have received prior radiation therapy, leading to changes in the immune microenvironment and disruption of the ecological niches for T cell storage (48). Consequently, there may be heterogeneity in the ecological niches among our patients, resulting in a non-significant comparison of the efficacy of combination therapy versus durvalumab monotherapy. There also exists a hypothesis suggesting that the standard dissection and/or functional excision of regional lymph nodes through surgical, radiotherapeutic, or a combined approach could potentially mitigate the impact of CTLA-4 inhibition in HNSCC (49), thereby resulting in a reduced effectiveness of tremelimumab. Furthermore, variations in the expression of PD-L1 may result in divergent outcomes in immunotherapeutic interventions. The KN040 investigation meticulously assessed the effectiveness of pembrolizumab versus the standard of care. Notably, within the subset manifesting the most substantial advantages from pembrolizumab, 25% of the patients exhibited PD-L1≥50%. In contrast, in the durvalumab-EAGLE cohort, only 28% of subjects demonstrated detectable PD-L1≥25% (50). Consequently, the heterogeneous sensitivity to ICIs among the patients could plausibly account for the absence of a statistically significant enhancement in efficacy when the combination therapy was compared with durvalumab monotherapy (49).

Similarly, the safety and tolerability of combination therapy were assessed. The analysis revealed that patients receiving combination therapy experienced a higher incidence of grade 3-4 trAEs and were more likely to discontinue treatment due to trAEs compared to patients receiving durvalumab monotherapy. There was no significant difference in terms of trAE-related deaths. A phase III clinical trial demonstrated that in patients with untreated unresectable, locally advanced, or metastatic uroepithelial carcinoma (51), the durvalumab plus tremelimumab treatment group exhibited a higher rate of grade 3-4 trAEs compared to the durvalumab treatment group (27% vs. 14%). This finding aligns with our analysis, indicating that combination therapies are associated with a higher incidence of side effects compared to monotherapy. In patients with R/M-HNSCC treated with ICI, the most common trAEs include diarrhea, anemia, Asthenia, and hypothyroidism (7, 20, 34). Therefore, regular and frequent monitoring for treatment-related complications is recommended for patients receiving ICIs, along with the implementation of individualized monitoring strategies in optimal scenarios.

The current study has several limitations. Firstly, only 3 randomized clinical trials are included in this study. Secondly, the prognosis and response to ICI therapy are influenced by various factors, including the TME composition, tumor cell immunogenicity, and extent of immune cell infiltration (52). Due to the absence of available data and other factors in the three included clinical trials, subgroup analyses could not be conducted. Moreover, it is important to note that open-label studies may introduce publication bias, particularly when conducted across multiple centers.

In the future, we anticipate an expansion randomized clinical trials with larger sample sizes and multi-center involvement, thereby facilitating more precise patient stratification. Additionally, the combination of immunotherapeutic agents with other modalities remains an area of significant interest (53). For patients with metastatic non-small cell carcinoma lung cancer (mNSCLC), the addition of durvalumab and four cycles of chemotherapy to a single course of tremelimumab demonstrates long-term survival benefits, early disease control, and manageable tolerability without compromising treatment exposure (54). Furthermore, the combination of durvalumab plus tremelimumab with proton therapy shows potential benefits for non-irradiated tumor lesions in heavily-treated patients with (55). Novel strategies based on ICI could be explored for the treatment of R/M-HNSCC.

## 5 Conclusion

The results of our meta-analysis indicated that while there was no statistically significant difference in efficacy between durvalumab monotherapy and combination therapy with durvalumab and tremelimumab, patients receiving the combination therapy may exhibit a higher ORR, albeit with a higher incidence of grade 3-4 trAEs. Close monitoring of adverse events is crucial when administering durvalumab with tremelimumab therapy to patients with R/M-HNSCC. Furthermore, our results could inform the design of future randomized controlled trials.

## Data availability statement

The raw data supporting the conclusions of this article will be made available by the authors, without undue reservation.

## Author contributions

XH: Writing – original draft. HZ: Conceptualization, Investigation, Writing – review & editing. KS: Methodology, Software, Writing – review & editing. JL: Formal analysis, Project administration, Writing – review & editing. WW: Methodology, Validation, Writing – review & editing. KL: Supervision, Writing – review & editing. ZY: Writing – review & editing, Funding acquisition, Resources.
